# Group II Intron-Encoded Proteins (IEPs/Maturases) as Key Regulators of Nad1 Expression and Complex I Biogenesis in Land Plant Mitochondria

**DOI:** 10.3390/genes13071137

**Published:** 2022-06-24

**Authors:** Ron Mizrahi, Sofia Shevtsov-Tal, Oren Ostersetzer-Biran

**Affiliations:** Department of Plant and Environmental Sciences, The Alexander Silberman Institute of Life Sciences, The Hebrew University of Jerusalem, Givat-Ram, Jerusalem 91904, Israel; ron.mizrahi1@mail.huji.ac.il (R.M.); sofia.shevtsov@gmail.com (S.S.-T.)

**Keywords:** intron encoded protein, IEP, maturase, MAT, group II intron, splicing, mitochondria, plant

## Abstract

Mitochondria are semi-autonomous organelles that produce much of the energy required for cellular metabolism. As descendants of a bacterial symbiont, most mitochondria harbor their own genetic system (mtDNA/mitogenome), with intrinsic machineries for transcription and protein translation. A notable feature of plant mitochondria involves the presence of introns (mostly group II-type) that reside in many organellar genes. The splicing of the mtRNAs relies on the activities of various protein cofactors, which may also link organellar functions with cellular or environmental signals. The splicing of canonical group II introns is aided by an ancient class of RT-like enzymes (IEPs/maturases, MATs) that are encoded by the introns themselves and act specifically on their host introns. The plant organellar introns are degenerated in structure and are generally also missing their cognate intron-encoded proteins. The factors required for plant mtRNA processing are mostly nuclearly-encoded, with the exception of a few degenerated MATs. These are in particular pivotal for the maturation of NADH-dehydrogenase transcripts. In the following review we provide an update on the non-canonical MAT factors in angiosperm mitochondria and summarize the current knowledge of their essential roles in regulating Nad1 expression and complex I (CI) biogenesis during embryogenesis and early plant life.

## 1. The OXPHOS System of Plant Mitochondria

Mitochondria are semi-autonomous organelles which act as the power-plans of eukaryotic organisms. Mitochondria are the sites of numerous metabolic processes that are essential for the cell. These include the oxidative phosphorylation (OXPHOS) system, which utilizes the electron transport from specific precursors of the citric acid cycle (i.e., NADH or FADH_2_), to generate a proton gradient needed for the phosphorylation of ADP into ATP, the primary carrier of cellular energy (reviewed by, e.g., [1,2]). The electron transport chain (ETC) is classically made up of four major protein complexes, the NADH:ubiquinone oxidoreductase (or complex I, CI), succinate dehydrogenase (CII), cytochrome bc1 complex (CIII), and cytochrome c oxidase (CcO, or CIV) that mediates the transfer of electrons to oxygen. Other components of the ETC include ubiquinone (UQ) that mediates the electron transport between CI / CII and CIII, and cytochrome c (cyt c), a small peripheral protein that transfers electrons from CIII to CIV. The respiratory system further relies on several carriers, which allow the transport of OXPHOS-related cofactors and substrates between the cytoplasm and the organelles. The mitochondria of plants, and a few other organisms, are also characterized by ‘non-energy’ conserving bypasses of the electron transport chain, which include the rotenone-insensitive type-2 NAD(P)H dehydrogenases (NDs) and alternative oxidases (AOXs) (reviewed by, e.g., [2]). The electrons transferred through the ETC from NADH (and other electron carriers) to O_2_ provide the chemical energy that is required to generate a membrane potential (ΔpH), by the pumping of H^+^ across the inner mitochondrial (cristae) membrane, which is coupled by the ATP-synthase enzyme (CV) to generate ATP. 

**Figure 1:**
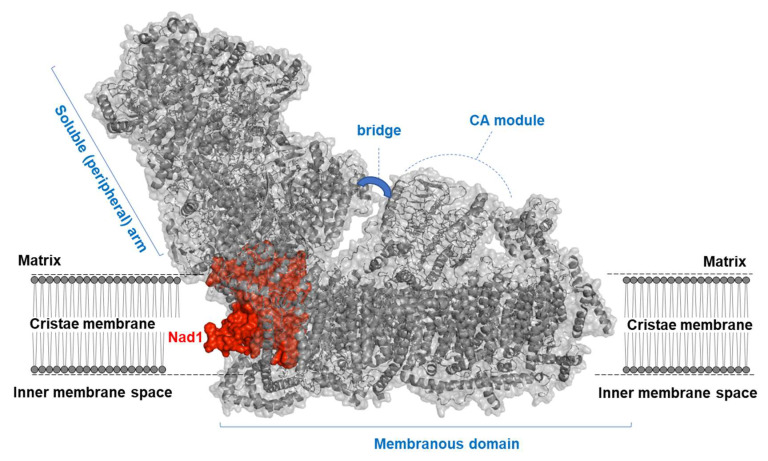
Structure of a plant mitochondria complex I. Complex I (NADH:ubiquinone oxidoreductase, or CI) is an L-shaped enzyme consisting of soluble arm (i.e., NADH oxidation domain) and membranous (i.e., proton translocation) domain. CI is illustrated here by a diagram of the ribbons within the surface of the holo-CI enzyme, based on the known cryo-EM structures of the complete complex I from plants [8,10]. The Nad1 subunit is shown in red, with the other mitochondrial CI subunits presented in gray color. The plant specific carbonic anhydrase (CA) module, which forms a characteristic mushroom-like structural extra domain, and the three subunits of the ‘bridge’ domain (i.e., B14, SDAP2, and C1-FDX) [10] are indicated in the figure.

The chief source of electrons for the ETC system is derived from NADH. These electrons enter the respiratory chain mainly via CI, the largest (about 1.0 MDa in size in plants) and most complex enzyme of the OXPHOS system (reviewed by, e.g., [3,4]). CI is a multimeric enzyme that forms an L-shaped structure, which is made of a ‘hydrophilic’ (soluble) arm and a ‘membranous’ domain (Figure 1) [5,6,7,8,9,10]. 

### 1.1. The Biogenesis of Respiratory Complex I

In animals, the mitochondrial respiratory CI is made of >40 different subunits, in which seven of these proteins are synthesized in the organelles by the mitochondrial ribosomes [5]. The plant CI enzyme is larger, consisting of between 47 to 51 subunits [8,10], nine of which are encoded in the mitochondria [11,12]. These include NADH dehydrogenase (CI) subunit1 (i.e., Nad1), Nad2, Nad3, Nad4, Nad4L, Nad5, and Nad6 that belong to the membrane domain (associated mainly with the pumping of proton across the cristae membrane), whereas Nad7 and Nad9 belong to the peripheral arm (mediates the transfer of electrons from NADH to UQ) [2]. The other subunits of CI are all nuclearly encoded and post-translationally imported into the organelles. The 14 CI subunits (i.e., Nad1, Nad2, Nad3, Nad4, Nad4L, Nad5, and Nad6 of the membrane domain, and the 24 kDa, 51 kDa, 75 kDa, Nad7, Nad9, PSST, and TYKY subunits of the soluble arm) are considered as the ‘core’ of CI, based on their high conservation in different organisms and as they compose the most compact form of CI enzyme (i.e., about 500 kDa in size) in some bacterial species [13]. The roles of other accessory subunits are less clear, but these subunits are generally postulated to play key roles in the assembly and biogenesis of the of holo-CI enzyme [14].

The structure and composition of the complete holo-CI from the model plant *Arabidopsis thaliana* has been recently reported [8]. The assembly of CI is a multifaceted process, which requires an orchestrated incorporation of different CI subunits encoded by two physically remote genomes, i.e., the nucleus and the mtDNA. Different approaches have been made to study the biogenesis of CI. These involved the disassembly of the native complex by detergent-treatments [15] and the analysis of mutants affected in the expression of various CI-subunits [14,16,17], as well as by the inhibition of mitochondrial translation [18]. Under limited expression, or the complete absence of a specific CI subunit (or an assembly factor) the holocomplex becomes unstable, leading to the accumulation of CI-assembly intermediates of lower molecular masses, which are apparent by separation of mitochondrial complexes under native conditions (see, e.g., [19]). Based on these data, and complexome-profiling assays [4,18,20], a modular assembly pathway was proposed for CI, in which intermediates of the hydrophilic and membranous domains are sequentially and separately formed and then assembled together into the native L-shaped holo-CI enzyme [14,17,18,21]. 

### 1.2. Plant Mitogenome Organization, Expression, and the Importance of mtRNA Metabolism during Embryogenesis

The mitogenomes of land plants display a high structural complexity, as compared to most other organisms [22]. Unlike their ”simpler” counterparts in Animalia, the expression of mitochondrial genes in plants requires extensive post-transcriptional RNA processing steps, such as trimming, editing (typically C-to-U in angiosperms), and the removal of many intron (mostly group II-type) sequences that reside in various CI genes (i.e., *nad1*, *nad2*, *nad4*, *nad5,* and *nad7*), but also interrupt the coding sequences of other essential protein-coding genes [23,24,25,26]. These processing steps are critical for the maturation of the pre-RNAs into functional mRNAs, which can be translated in the organelle into the protein they encode. Mutants affected in the expression or processing of mitochondria-encoded subunits often display arrested embryo development and altered growth and developmental phenotypes [24,27,28,29]. In the following review sections, we discuss some recent findings related to the roles of plant-related intron-encoded proteins (i.e., IEPs/maturases) in regulating the splicing of mitochondrial *nad* genes, and hence in the biogenesis of the OXPHOS system, during embryogenesis and early plant life. 

In summary, the mitochondrial NADH dehydrogenase enzyme (CI) is composed of many different subunits (47 subunits in Arabidopsis [10]) that are encoded by both the nuclear and organellar genomes. Genetic and biochemical data show that the biogenesis of CI is an intricate process that involves complex regulatory steps, whichare most likely required to coordinate the mitogenome expression with the expression, import, and assembly of the nuclear-encoded subunits. A notable feature of plant mitochondria involves the presence of numerous group II introns that reside in the coding regions of many essential genes (in particular within *nad* genes), which their splicing is essential for the production of functional mature messenger RNAs (mRNAs). In the following section we review the current knowledge on group II intron splicing and how this essential step in organellar gene expression may relate to the ability of land plants to regulate their respiratory activities in response to developmental or environmental signals. 

## 2. Group II Introns: Remnants of a Primordial RNA World Which Have Invaded into the Coding Sequences of Many OXPHOS-Encoding Genes in Plant Mitochondria

**Figure 2:**
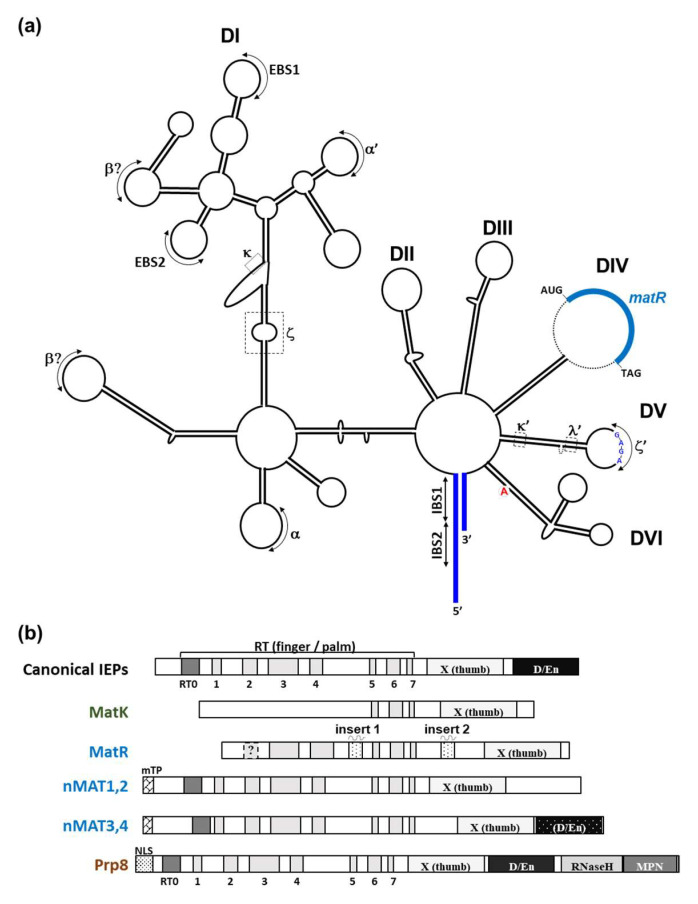
Schematic representation of the secondary structure of plant group II intron and IEP proteins. (**a**) Secondary structure model of *nad1* intron 4 (*nad1* i4) with its maturase-related (MatR) open reading frame. The six subdomain DI and DII, DIII, DIV, DV, and DVI are outlined within the structure. The different tertiary interaction sites (including the GNRA tetraloop inside DV) of group II introns are indicated by roman letters, together with the exon-intron (EBS1:IBS1, or EBS2:IBS2) binding sites and the canonical bulged-A residue found within DVI. The putative secondary structure of *nad1* i4 was generated by the Alifold [40] Mfold [41] servers and the predicted secondary structure of Arabidopsis *nad1* i4 [42]. (**b**) Schematic representation of bacterial and plant IEPs/MATs and the spliceosomal Prp8 protein. Shaded boxes represent different domains associated with model group II intron-encoded maturases and the nuclear Prp8 protein, in accordance with previous reports [34,42]. These include the conserved RT domain of group II introns (pfam-00078), with the intrinsic N-terminal RT0 and the seven (RT 1-7) finger and palm motifs, the X (thumb) domain (pfam-08388) subdomain, and in some cases a DNA binding and endonuclease (D/En) subdomain (pfam-01348) found in the C-terminal part of the protein. MatR harbors two specific insertions found between RT4 and RT5 [42] and between RT-7 and domain X. nMAT1-to-4 further contain a mitochondrial localization signal peptide (mTP) region, while Prp8 harbors a nuclear localization signal (NLS), located at the N-terminal regions of the proteins.

Different RNAs, such as ribozymes, retrotransposons, or self-splicing introns (i.e., group I or II) act as chemical catalysts, capable of performing chemical reactions in the absence of any proteinaceous cofactors [30]. RNA molecules that belong to the group II-class (Figure 2a) are comprised of the ribozyme (intron or retrotraposone element), and in some cases also an intron-encoded protein (i.e., IEP or maturase, MAT) (Figure 2b). It is generally assumed that the chemistry of the splicing reaction(s) is RNA-mediated, while the IEPs aid with the folding of their cognate introns under physiological conditions in vivo [31,32]. Accordingly, model introns in this class can self-splice in the absence of their MATs (or any other proteinaceous cofactors) under non-physiological (e.g., high salt or high temperature) conditions in vitro [31,32]. In addition to their roles in splicing, the MATs were further shown to enable the transposition (retrohoming) of their cognate introns, via the ‘reverse-splicing’ of the excised intron RNA into a DNA target site. Structural analyses provided important insights into the structures and functions of group II intron RNAs and their splicing and homing MAT cofactors [33,34]. These features are discussed in more details in Section 3, below.

Based on parallels in the structures and the splicing chemistry between group II introns and the nuclear spliceosomal introns, it was hypothesized that the two intronic groups share a common evolutionary history. This was further supported by various data that showed that the spliceosomal core protein, Prp8, seems to be evolutionary and functionally related to group II MATs [35]. The phylogeny of group II introns in prokaryotes and in the organelles of eukaryotes [31,32,36] indicate that the introns probably originated in eubacteria, and then were introduced to the eukaryotes via endosymbiotic gene transfer (reviewed by, e.g., [29,37,38,39]).

Although group II RNAs are not conserved in their primary sequences, they all share a similar fold of six helical domains (DI-VI) that radiate from a central hub (Figure 2a). Some of the introns also harbor an open reading frame (ORF) encoding a MAT protein that resides within D4 (Figure 2a,b) [43,44]. The six intronic domains further form internal long-range interactions (indicated by Greek letters or EBS/IBS, the exon binding sites and intron binding sites; Figure 2a), which are pivotal for the folding of the intron into a functional tertiary structure, presumably to position key nucleotides at the active site [31,32,33]. Sequences that are related to group II introns are in particular plentiful in the organellar genomes of land plants [31,32,37,38], although the chromosomes of some bacteria may host numerous group II sequences [36]. In eubacteria, the introns are often found near genes or after *rho* elements and are therefore expected to have a minor effect on gene expression. By contrast, in angiosperm mitochondria, group II introns are predominantly found in protein-coding genes (Table 1) [11,12], where they are particularly prevalent within genes encoding various NAD subunits, but where they also interrupt the coding regions of a few genes encoding cytochrome c biogenesis (*ccm*), cytochrome c oxidase (*cox*), or ribosomal-related subunits. 

The majority of the plant organellar introns are degenerated, such as they have lost various elements that are considered to be essential for splicing and are also generally lacking the related *mat* genes [38]. Moreover, several mitochondrial introns in different land plants are further fragmented into individually-transcribed intron segments, so they must assemble in *trans*. In *Arabidopsis thaliana*, these include *nad1* introns 1 and 3, *nad2* intron 2, and *nad5* introns 3 and 4 (Table 1), although fragmentation of other mitochondrial group II introns (e.g., *nad1* intron 4 or *cox2* intron 1) is also apparent in other plant species [38,45]. The *trans*-splicing activity is typically bipartite in structure, in which the fragmentation sites of the group II intron occur within the intron D4, either upstream or downstream of the *mat* gene. Intriguingly, the *trans*-splicing activity of plant organellar group II introns may also relate to the interactions between different snRNAs of the spliceosomal machineries in the nuclear genomes of eukaryotes [29]. 

Taken together, the splicing of the *cis*- or *trans*-introns in plant mitochondria is crucial for the biogenesis of respiratory machinery and relies upon the activities of different subsets of splicing cofactors. In the following section we discuss the roles of MATs, and their accessory splicing factors, in the processing and maturation of organellar pre-RNAs, and hence in regulating the biogenesis of the respiratory system in the mitochondria of land plants.

## 3. Maturases (IEPs) as Key Regulators of Gene Expression in Angiosperm Mitochondria

As descendances of a primordial RNA world [46,47,48], the splicing reaction of model group II introns can be facilitated in vitro, in the absence of any protein cofactors. Yet, for their efficient splicing in vivo, these introns need to form interactions with various protein cofactors. The group II splicing factors can be classified into two distinct groups: (a) MATs that are evolutionary related to their group II intron hosts, and (b) other ‘*trans-acting*’ factors that belong to different RNA-binding protein families, which were probably recruited during evolution to function in the splicing of group II introns. Canonical group II MATs are characterized by a few functional motifs that are required for the splicing or intron mobility functions: an N-terminal domain that is related to a viral-type reverse transcriptase (RT) (pfam 00078), harboring the fingers, palm, and a thumb (X, pfam 08388) subdomains, which are followed in some MATs by a C-terminal DNA binding and endonuclease (D/En, pfam 01348) domain (Figure 2b). The best characterized maturase to-date is the *Lactococcus lactis* LtrA protein that is encoded by the *ltrB* intron. As other model group II introns, *ltrB* is capable of catalyzing its own excision under high temperature and salt conditions, in vitro [31]. The splicing of *LtrB* under physiological conditions relies on its cognate LtrA maturase, which was shown to bind with high affinity and specificity to its host RNA [49,50,51]. The association of LtrA with stem-loop regions found within DIV, as well as with DI, DII, and DVI, seem to be essential for the folding of *ltrB* intron into its catalytically active form, as well as to enable the ‘retrohoming’ of the *LtrB* [31,52]. The postulated roles of MAT factors in facilitating the splicing of their cognate introns by stabilizing short- or long-distance interactions, which seem indispensable for the intron folding, are also supported by structural analyses of intron-bound MAT proteins [33,34]. However, what kind of roles play the atypical plant-encoded MAT proteins in the splicing (or homing) of group II introns in plant organelles? Seemingly, the plant mitochondrial introns have diverged considerably from their eubacterial ancestors [23,31,32,37,38]. These are often lacking regions that are conserved among model self-splicing introns (and are thus considered to be essential for the splicing reaction) and are generally also missing their related IEP genes [29,32,37,38,53]. In this review, we summarize recent advances in our understanding of the role of maturase-related proteins in plants.

### 3.1. Organelle-Encoded MAT Proteins

The plastids harbor a highly conserved maturase ORF, named MatK, which is encoded by the single intron in *trnK* gene [54]. The *matK* gene encodes a degenerated IEP that was found to be associated with multiple pre-mRNA targets (a subset of group IIA introns) in vivo [55,56,57]. The number of IEPs seems to be more variable in the mitogenomes of land plants, with the higher number of IEP sequences found in the mtDNAs of liverworts [31,42,58] thatare regarded as the most basal group of land plant species [59]. Notably, several of the organellar introns that lack a functional ORF, contain remnants of IEP-related sequences in D4, thus suggesting a gradual of loss of IEPs throughout the evolution of land plants [32]. In angiosperms, only a single maturase has been retained in the mitochondria, the *matR* gene encoded by the fourth intron of *nad1* gene (i.e., *nad1* intron 4, or *nad1i728*) [60]. MatR harbors a truncated RT domain, and possibly fragments of the D/En motifs [31,42]. Its high conservation among different angiosperm species, as well as RNA editing events that restore evolutionarily conserved amino acid sequences, suggest that *matR* encodes a functional splicing cofactor that is vital for normal mitochondrial activities, but most likely lacks the RT and intron-homing functions associated with canonical IEPs [29,32,42,55,56,57,61,62,63]. In addition to the indirect observations, genetic and biochemical data have further provided more direct evidence for a role for MatR in the splicing of many mitochondrial introns [42]. 

### 3.2. Nuclear-Encoded Maturases in Plants (nMATs)

A hallmark of the organellar group II splicing cofactors in plants is that they are mostly nuclear-encoded. In addition to the two organelle-encoded MATs, land plants also contain several IEP-related proteins that were transferred as functional genes into the nucleus during the evolution of land plants [53,64]. In the model plant *Arabidopsis thaliana* these include four nuclear genes, which are annotated as nMAT1-4 (Table 2), all of which are forecast to harbor organellar localization signals in their N-terminal regions [53,64]. The TAIR database specifies the presence of two possible introns in one of these genes, i.e., *nMAT3* (*AT5G04050*), that their splicing would lead to a degenerated IEP product, which harbors a truncated RT and lacks the X and D/En domains. However, the splicing of these introns cannot be validated by experimental (i.e., RNA seq or RT-PCR) data [65].

Based on their phylogeny and deduced amino acid sequences the four nMATs are further divided into two IEP classes [53,64]: nMAT1 and nMAT2 that have both lost the D/En motif at their C-terminal regions, whereas nMAT3 and nMAT4 have retained an aberrant En/D domain, with various mutations that are expected to restrict endonuclease activity. In agreement with the in-silico data, GFP localization assays showed that the four nMATs in Arabidopsis are all located in the mitochondria, in vivo [64]. Genetic studies further indicated that the nMATs function in the splicing of different subsets of mitochondrial group II introns in angiosperms, where they found to serve as key factors in the maturation of *nad* pre-mRNAs in Arabidopsis mitochondria (Table 2). The homozygote *nmat* mutant lines show altered development and retarded growth phenotypes, modified respiration functions, and altered stress responses, which are tightly corelated with respiratory CI biogenesis defects [24,25,27].

nMAT1 is pivotal for the splicing of *nad1* intron 1, *nad2* intron 1, and *nad4* intron 2 [66,67], while nMAT2 affects the splicing efficiencies of many intron targets, but seems in particular important for the processing of *cox2* intron 1, *nad1* intron 2, and *nad7* intron 1 [64,68]. Notably, *nad1* intron 2, *nad7* intron 1, and *cox2* all seem to be lacking the canonical bulged A residue in D6, which is required for the first *trans*-esterification step and the release of the 5′ exon [64]. The specific roles of nMAT2 in mitochondrial group II intron splicing has not yet been established, but an intriguing possibility is that nMAT2 may function in the hydrolysis of the phosphodiester bond at the 5′ splice site, or in recruiting specific nucleases required for the release of the 5′ exon [37,64]. Our genetic analyses indicated that nMAT3 [65] and nMAT4 [69] also play key roles in *nad1* maturation, by promoting the splicing of the *trans*-spliced *nad1* introns 1 and 3 and the *cis*-spliced *nad1* intron 4 (Table 2). The lack of redundancy of nMAT3 and nMAT4 in the splicing of the same intron targets is interesting, as biochemical studies have indicated that model IEPs form a dimer with their cognate group II intron RNA targets, in vitro [76,77]. Likewise, the expressed IEPs’ RT domain were also shown to form dimers in solution [78]. However, the dimerization of model IEPs was not observed in cryo-EM analyses of model introns bound by their specific IEPs [34]. It is therefore remains possible that the atypical nuclear-encoded IEPs, nMAT3 and nMAt4, may form a heterodimer complex with their RNA targets in plant mitochondria [65,69]. Biochemical analyses that aim to address the association between nMAT3, nMAT4, and their genetically identified intron target, such as *nad1* intron 3, are currently underway in our laboratory.

### 3.3. MAT Accessory Factors in Plant Mitochondria

Genetic analyses have revealed that in addition to MatR and the four nMATs, the splicing of the complete set of mitochondrial group II introns relies on additional nuclear-encoded protein cofactors (reviewed by, e.g., [23,24,25,37]). These characteristically belong to a diverse set of RNA-binding protein families, such as RNA helicases (Pfam 01348), mTERFs (mitochondrial transcription and termination factors, Pfam 02536), PORR-related (plant organelle RNA recognition domain, Pfam 11955) cofactors, and several proteins which contain the conserved PPR domain (pentatricopeptide repeat motif, Pfam 13812). While the MATs, RNA helicases [72,73,79], and CRM proteins [74] affect the splicing of multiple mitochondrial intron targets, the PPRs, mTERF [80], PORR [81], UL18 [82], or RCC proteins [83] seem to be more specific, influencing the splicing of a single or only a few group II introns. Currently, there is only a little mechanistic information about the molecular functions of the plant organellar splicing factors, in what way they enable the processing of their intron RNA targets and whether these may cooperate together in the splicing reaction [23,24]. We speculate that the recruitment of *cis*- and *trans*-acting cofactors was correlated with the degeneration of the organellar introns in plants, i.e., the need to stabilize secondary or tertiary interactions in the RNAs, or maybe in some introns also to facilitate the trans-esterification steps that lead to the release of the 5′ exon, a prerequisite for the joining of the exons. 

### 3.4. Mitochondrial RNA Metabolism and Group II Intron Splicing Are Vital Processes for Successful Embryogenesis, Germination, and Early Plant Development

Plants exhibit complex cellular signaling between their organelles and the nucleus, thus allowing them to manage the specific energy requirements of the cell during particular growth and developmental stages [84]. These processes involve alterations in cellular energy metabolism, as well as controlling the biogenesis and maintenance of the mitochondria. Early studies indicated that increased respiration is followed by the expression of proteins involved in electron transport, the TCA cycle, ATP synthesis, and other metabolic activities that are essential for the plant [85]. Biochemical and genetic studies further show that mitochondrial proteins that are essential for the biogenesis of the respiratory system gradually increased in their abundances during imbibition and early stages in plantlet establishment, showing the highest abundances in the mature seedlings [42,86,87].

The organellar gene-expression machineries and respiratory system require the assembly of subunits encoded by both nuclear and mitochondrial genomes. Various databases such as ‘The Arabidopsis Information Resource’ (TAIR) and ‘Genevestigator’ [88] indicate to a differential expression of genes encoding MatR [42], nMAT1-4 [64,65,67,69], and various other nuclear-encoded mitochondrial splicing cofactors [89], with their mRNAs levels being dominant in embryonic organs, during germination and at seedling establishment [84,86]. Factors that regulate the maturation of primary (non-functional) mitochondrial transcripts are assumed to play central roles in the regulation of organellar biogenesis during key developmental stages, such as in embryo development and seed development, germination, and early plant life [42,86,87]. We previously proposed that complex mtDNA structures with a highly regulated mode of gene expression (e.g., the splicing of introns that reside in many essential genes) may provide land plants with better means to regulate mitochondrial functions and cellular energy metabolism in response to different endogenous and environmental signals, and in particular during embryogenesis and germination [84].

### 3.5. From Specific Mono-Intronic Maturase-Facilitated Splicing in Bacteria towards More General ‘Proto-Spliceosomes’ in the Mitochondria of Angiosperms

The analysis of MATs and various other factors involved in the splicing of group II introns have provided important insights into the roles of RNA-binding cofactors in mitochondria gene-expression and the biogenesis of the respiratory system during early plant life [27,84]. Noticeably, group II introns have been greatly multiplied in numbers in the mitogenomes (and plastid DNAs) of land plants. Yet, these have retained only a single, or a few genes encoding an intron maturase in each organelle [29,31,32,37]. Genetic and biochemical studies have indicated that not only do the plant MATs show sequence deviation from their ancestral intron-specific IEPs in bacteria, but are also remarkable by acting in the maturation of multiple intron targets. The plant MATs were further found to affect the splicing of organellar introns together with additional factors, thus acting as organellar proto-spliceosomes, which make them interesting and potentially models for the early evolution of nuclear spliceosomal splicing [29]. Yet, it remains unclear how a specific mono-intronic maturase has evolved into a general splicing cofactor that affects the splicing of several intron targets. Deviations in the RT region may be associated with the loss of intron specificity.

Canonical IEPs harbor seven conserved motifs (RT1-7) that are related to RTs, but also harbor an additional subdomain (RT0) found in the most N-terminal region of group II maturases, and a thumb (X) motif that is followed in some cases by a DNA binding and endonuclease (D/En) domain (Figure 2b) [31,42,90]. The classical RT subdomains and the D/En motif of model IEPs are linked to the intron mobility (retrohoming) activities, the X (thumb) domain is associated with splicing [34]. Different data suggest that the RT0 subdomain may provide with sequence specificity, which is possibly mediated by the interaction of positively charged amino acids in the RT0 with regions in D4 of the intron RNA [34]. 

**Figure 3:**
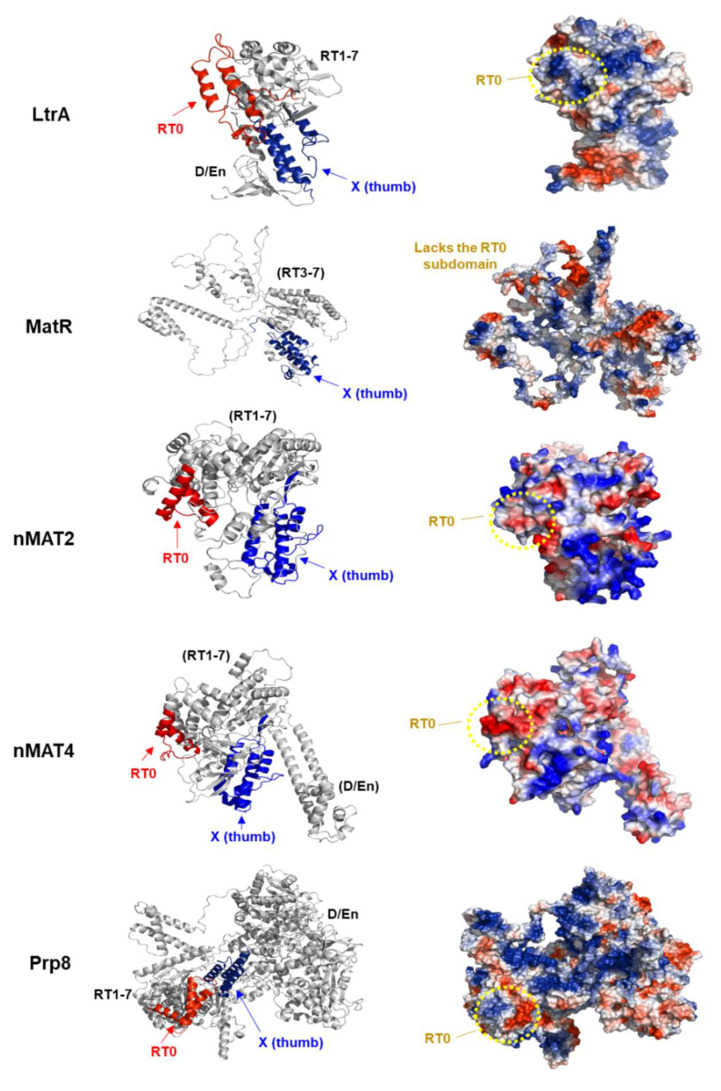
Structural modeling of plant maturase-related proteins. To get more insights into the plant maturase mode of action, in particular of RNA recognition, we used the atomic 3D models of the Arabidopsis MATs available at the AlphaFold server [91]. The proposed folds of MatR and the four nMAT proteins were visualized by the PyMol package [92]. MatR (PDB AF- A0A2P2CLH3) harbors a truncated RT motif, which lacks the RT0 motif, and an intact X (thumb) domain. The nuclear-encoded mitochondria-localized nMAT2 (PDB AF-Q9FJR9) protein has a degenerated RTs and lacks the D/En, while nMAT4 (PDB AF- Q9CA78) contains degenerated RT and D/En motifs [53,68]. The known structures of LtrA (PDB 5G2X) [93] and the spliceosomal Prp8 protein (PDB 5LJ3) [94] are shown in the upper and lower panels, respectively, and illustrated by a ribbon diagram (**left panels**) and by a cartoon with the surface of each protein (**right panels**). Red color in the ribbon diagram indicates the RT0 subdomain, while the X domain is labeled in blue. Red color on the solvent-accessible surfaces of the IEP/MAT or Prp8 proteins (**right panels**) indicates negative values (i.e., negatively-charged residues), blue indicates positive values (basic amino acids), whereas white color shows near zero values. The positively charged surfaces are anticipated to indicate RNA binding and recognition sites. The RT0 regions of LtrA are positively charged, while the RT0 surfaces of nMAT2, nMAT4, and Prp8 are mostly negatively charged.

Figure 3 provides a representation of the established structures of a canonical IEP (i.e., LtrA, PDB 5G2X), the spliceosomal Prp8 factor (PDB 5LJ3) and the predicted 3D folds (AlphaFold server) [91] of three Arabidopsis mitochondrial IEPs, i.e., MatR (PDB AF- A0A2P2CLH3; harbors only an intact X (thumb) domain, and a truncated RT motif that lacks the RT0 subdomain), nMAT2 (PDB AF-Q9FJR9; both nMAT1 and nMAT2 have degenerated RTs and lack the D/En,), and nMAT4 (PDB AF- Q9CA78; nMAT3, and nMAT4 harbor degenerated RT and D/En motifs). The plant MATs harbor an intact X (thumb) domain but seem to contain degenerated and non-functional RT and D/En domains. Notably, the *matK* or *matR* genes are both missing the N-terminal region (contains the RT0 subdomain) of model group II MATs (Figure 2b). In addition, the RT0 subdomains of the four nMATs seem degenerated, such as they lack various basic amino acids that are expected to assist in the establishment of specific intron RNA interactions [34]. 

In summary, canonical maturases (IEPs) are known to bind with high affinity and specificity to their own ‘host’ intron RNAs. Yet, their related homologs in angiosperms (i.e., MatR and the four nMATs) were found to act in the splicing of multiple pre-RNA targets [29]. The ability of the plant MATs to function in the splicing of different organellar introns could relate to the degeneracy of their RT0 regions, which lack the typical basic surface (Figure 3). Similarly, the loss of basic residues in the RT0 subdomain of the core spliceosomal factor, Prp8, may relate to its ability to act in the splicing of a large subset of pre-mRNA targets in the nucleus of eukaryotic organisms [34]. These data provide with stimulating clues into the molecular basis of splicing and gene structure evolution. It is expected that MATs were already present in the introns of the ancestral symbiont of the mitochondria. Variations in the exon–intron structures of genes involved the loss or gain of intron sequences, which could have marked influences on gene expression levels and patterns (e.g., altered mRNA levels, as well as different mRNA species generated by alternative splicing). During evolution, the group II introns have multiplied in the mitogenomes of land plants, while being inserted into different protein-coding genes, most of which reside in genes encoding different NADH dehydrogenase (Nad) subunits (see Section 4). Some of the *nad* introns (in *Arabidopsis thaliana* these include *nad1*, *nad2,* and *nad5*) are fragmented and excised in *trans*, in which the different exons are spliced from different RNA molecules. The extra complexity in the expression of *nad* genes may have arisen in plants as a means to provide a tighter regulation of mitochondria gene expression, or possibly to enhance mRNA accumulation [84,95]. In accordance with the complexity of plant mitochondria gene expression and mtRNA metabolism, the majority of the IEPs in land plants were transferred to nuclei as self-standing ORFs that contain mitochondria localization signals, while their activities have been assisted by various nuclear-encoded accessory splicing cofactors. In Section 4 we discuss the pivotal roles of group II intron splicing cofactors in the regulation of Nad1 expression and CI biogenesis in plants.

## 4. Nad1 as a Crossroad for Complex I Assembly in Animal and Plant Mitochondria

Complex I (CI) serves as a major entry point for electrons from NADH into the respiratory chain. It is also the largest (about 1000 kDa in plants) and most complex membranous enzyme of the OXPHOS system. The biogenesis of CI in plants relies on extensive post-transcriptional RNA processing steps (i.e., trimming, editing, and *cis*- or *trans*-splicing) which take place in the mitochondria. The RNA processing events in plant mitochondria are mediated by different RNA-binding cofactors, which are essential for the maturation of the organellar pre-mRNAs into functional (translatable) mRNAs. Notably, the five IEPs in Arabidopsis mitochondria (i.e., MatR and nMAT 1- 4) are all involved in the excision of at least one of the four introns found within the three different *nad1* pre-mRNA transcripts. Genetic analyses revealed that the maturation of Nad1 subunit in Arabidopsis mitochondria involves at least 11 different splicing cofactors (Table 2). In addition to MatR [42], the four nuclear-encoded nMATs 1-to-4 [64,65,66,67,68,69], these also include two PPR proteins (i.e., MISF74 [70] and OTP43 [71]), two RNA helicases (ABO6 [72] and PMH2 [68,73]), a CRM protein (mCSF1) [74], and the RAD52-like (ODB1) protein [75], which further emphasize the extra complexity of Nad1 expression in land plant mitochondria. 

Genetic and biochemical data suggest that Nad1 expression is determinantal for the assembly of the membrane arm in plants, e.g., by assisting in the recruitment of different CI subunits or in the stabilization of sub-CI intermediates. The assembly of the membrane arm in animals begins with ND1 (the ortholog of Nad1 in plants), making it the first mitochondria-encoded NAD subunit in CI assembly [21]. Likewise, in plants, the loss of Nad1 results in CI biogenesis defects and in many cases also the accumulation of non-functional CI-intermediates of low molecular masses [17,65,69,70,71]. Hence, the processing of the pre-*nad1* RNA transcripts is a prerequisite for respiratory CI assembly and the biogenesis of the OXPHOS system in angiosperms. Maturases and their accessory splicing cofactors in plant mitochondria seem to act as major regulators for Nad1 expression, and thus control the biogenesis of respiratory CI and the OXPHOS system in land plant mitochondria. We speculate that the degeneration, and ultimately the loss, of IEPs from the host introns was enabled once the plant MATs (i.e., MatR and the nMATs) have evolved so that they can function in the splicing of multiple intron RNA targets, as well as the acquisition of other RNA-binding cofactors that have been recruited from the host genome to function in mitochondrial group II intron splicing [29]. Importantly, these factors have the ability to provide the plant cells with a means to regulate mitochondrial functions in accordance with developmental and environmental signals (see also [84]).

## Figures and Tables

**Table 1:** List of group II intron-containing genes in *Arabidopsis thaliana* mitochondria.

Group II Intron	Synonym	Name of Protein	Intron Type	
*ccmFc* intron 1	*ccmFCi829*	Cytochrome c maturation protein Fc	cis-splicing	
*cox2* intron 1	*cox2i691*	Cytochrome c oxidase subunit 2	cis-splicing	*
*nad1* intron 1	*nad1i394*	NADH dehydrogenase subunit 1	trans-splicing	
*nad1* intron 2	*nad1i477*		cis-splicing	*
*nad1* intron 3	*nad1i669*		trans-splicing	
*nad1* intron 4	*nad1i728*		cis-splicing	**
*nad2* intron 1	*nad2i156*	NADH dehydrogenase subunit 2	cis-splicing	
*nad2* intron 2	*nad2i542*		trans-splicing	
*nad2* intron 3	*nad2i709*		cis-splicing	
*nad2* intron 4	*nad2i1282*		cis-splicing	
*nad4* intron 1	*nad4i461*	NADH dehydrogenase subunit 4	cis-splicing	
*nad4* intron 2	*nad4i976*		cis-splicing	
*nad4* intron 3	*nad4i1399*		cis-splicing	
*nad5* intron 1	*nad5i230*	NADH dehydrogenase subunit 5	cis-splicing	
*nad5* intron 2	*nad5i1455*		trans-splicing	
*nad5* intron 3	*nad5i1477*		trans-splicing	
*nad5* intron 4	*nad5i1872*		cis-splicing	
*nad7* intron 1	*nad7i140*	NADH dehydrogenase subunit 7	cis-splicing	*
*nad7* intron 2	*nad7i209*		cis-splicing	
*nad7* intron 3	*nad7i676*		cis-splicing	
*nad7* intron 4	*nad7i917*		cis-splicing	
*rpl2* intron 1	*rpl2i846*	60S ribosomal protein L2	cis-splicing	
*rps3* intron 1	*rps3i74*	40S ribosomal protein S3	cis-splicing	

* Lack a canonical bulged A residue. ** The intron encodes a conserved IEP, the MatR maturase.

**Table 2:** List of *nad1* splicing factors in *Arabidopsis thaliana* mitochondria.

		*nad1* *				
Splicing Factor	Protein Family	i1	i2	i3	i4	Ref’s
MatR	IEP/Maturase					[42]
nMAT1	IEP/Maturase					[66,67]
nMAT2	IEP/Maturase					[64,68]
nMAT3	IEP/Maturase					[65]
nMAT4	IEP/Maturase					[69]
MISF74	PPR					[70]
OTP43	PPR					[71]
ABO6	RNA helicase					[72]
PMH2	RNA helicase					[68,73]
mCSF1	CRM					[74]
ODB1	RAD52-like					[75]

* White boxes indicate introns of which their splicing does not rely on the specific protein factor. Grey boxes indicate that the factor is involved, but not necessary, for splicing, while a black box indicates that the specific factor is essential for the splicing activity.

## Data Availability

Data is contained within the article.
